# Hysteroscopy as a Primary Tool in Exploration and Treatment of Infertility: Single Center Experience in Western Romania

**DOI:** 10.3390/diagnostics11101917

**Published:** 2021-10-16

**Authors:** Cosmin Citu, Florin Gorun, Andrei Motoc, Ioan Sas, Oana Maria Gorun, Bogdan Burlea, Denis Mihai Serban, Radu Neamtu, Ioana Mihaela Citu

**Affiliations:** 1 Department of Obstetrics and Gynecology, “Victor Babes” University of Medicine and Pharmacy Timisoara, 2 Eftimie Murgu Sq, 300041 Timisoara, Romania; citu.ioan@umft.ro (C.C.); sasioan56@yahoo.com (I.S.); denis.serban@gmail.com (D.M.S.); neamtu.radu@umft.ro (R.N.); 2 Department of Anatomy and Embryology, “Victor Babes” University of Medicine and Pharmacy Timisoara, 2 Eftimie Murgu Sq, 300041 Timisoara, Romania; amotoc@umft.ro; 3 Department of Obstetrics and Gynecology, Municipal Emergency Clinical Hospital Timisoara, 1-3 Alexandru Odobescu Street, 300202 Timisoara, Romania; oanabalan@hotmail.com (O.M.G.); bogdanburlea@yahoo.com (B.B.); 4 Department of Internal Medicine I, “Victor Babes” University of Medicine and Pharmacy Timisoara, 2 Eftimie Murgu Sq, 300041 Timisoara, Romania; citu.ioana@umft.ro

**Keywords:** hysteroscopy, infertility, intrauterine abnormalities

## Abstract

(1) Background: Infertility is a disease that affects millions of individuals worldwide. Intrauterine lesions are common in infertile women, hysteroscopy being considered the gold standard for assessing them, even if in routine clinical practice indirect imaging techniques are the first-line investigative tools. The aim of the study was to evaluate hysteroscopic findings among women with unexplained infertility and to analyze fertility outcomes after operative hysteroscopy; (2) Methods: a retrospective cohort study was conducted among 198 women with infertility that had undergone hysteroscopy as the first step of their infertility workup. (3) Results: The median age of the participants was 34 years, 67.7% of them being diagnosed with primary infertility. The most common abnormalities were endometrial polyps, uterine synechiae and uterine fibroids. In addition, pregnancy rates were 23.1% after hysteroscopic polypectomy, 11.1% after hysteroscopic myomectomy and 23.8% after uterine synechiae resection; (4) Conclusions: Endometrial polyps were the most common uterine abnormality found in women with infertility. Hysteroscopic interventions appeared to increase pregnancy rates and outcomes among these women.

## 1. Introduction

Infertility is a disease defined by the failure to achieve a pregnancy after 12 months or more of regular, unprotected sexual intercourse. Worldwide, 48 million couples and 186 million individuals live with infertility [1]. Globally, the prevalence rate of female infertility increased by 14.9% from 1366.85 per 100,000 in 1990 to 1571.35 per 100,000 in 2017 [2]. Estimates suggest that female infertility was the cause in 37% of infertile couples. In the female, infertility may be caused by tubal disorders, uterine disorders, disorders of the ovaries or disorders of the endocrine system [1]. In Romania, 16.8% of the studied fertile population was or is in a situation of infertility, according to a study by the Romanian Human Reproduction Association [3]. 

Intrauterine lesions can interfere with spontaneous fertility, being common in infertile women (40–50%). Furthermore, these lesions can compromise pregnancy rates in assisted reproduction [4]. Uterine factors associated with infertility include endometrial polyps, leiomyomas, müllerian anomalies or synechiae [5]. Although hysteroscopy is not commonly used for the initial assessment of women with infertility, direct visualization of the uterine cavity provides the most definitive method for diagnosing endometrial polyps, uterine synechiae or submucosal fibroids [5]. Thus, the hypothesis is that uterine abnormalities found on hysteroscopy are common in infertile women, operative hysteroscopy being beneficial in such cases even if the evidence is low.

In Romania, according to Romanian Human Reproduction Association, the main causes of female infertility are fallopian tube disorders (23%), uterine factors (22%), endometriosis (17%) and a reduced ovarian reserve (15%) [3]. The aim of this study is to evaluate hysteroscopic findings among women who were attending our second-degree medical care unit with unexplained infertility. We also propose analyzing fertility outcomes after operative hysteroscopy.

## 2. Materials and Methods

### 2.1. Study Design, Settings and Participants

A retrospective cohort study was conducted among women who had undergone hysteroscopy as part of their unexplained infertility workup at the Obstetrics and Gynecology Clinic of the Timisoara Municipal Emergency Hospital between January 2018 and December 2020. The study was approved by the Ethics Committee of the “Victor Babes” University of Medicine and Pharmacy (Timisoara, Romania, approval no. 6664/15 June 2020) and by the Ethics Committee of the Timisoara Municipal Hospital (approval no. I-15505/15 June 2020).

The Obstetrics and Gynecology Clinic of the Timisoara Municipal Emergency Hospital is a university medical unit from Timisoara, Romania. Our clinic is the largest obstetrics and gynecology unit in the western part of Romania. The reproductive age women population of this area, according to the national census, is 443.473 persons.

The included participants had met the following criteria: (1) be at reproductive age (between 18–49 years old); (2) diagnosed with infertility (failure to achieve a pregnancy after 12 months or more of regular unprotected sexual intercourse); (3) have undergone a diagnostic hysteroscopy. The follow-up was performed by telephone. The following information was recorded: (1) number of pregnancies; (2) time between intervention and pregnancy; (3) pregnancy outcomes.

### 2.2. Procedure

Transvaginal ultrasound was performed in all patients for the initial evaluation of the uterus, ovaries and adnexa, as well as for the evaluation of the ovarian reserve by counting the antral follicles. All 198 participants underwent diagnostic hysteroscopy under general anesthesia and in sterile conditions. Hysteroscopy was performed in the operating room, office hysteroscopy not being available in our clinic. All women signed an informed consent form before undergoing the procedure. A detailed explanation of the procedure was given by the operating physician in the operative registers and archived in the hospital’s database. Diagnostic hysteroscopy was performed using a 2 mm diameter continuous-flow endoscope. The bilateral tubal ostia were identified at the beginning of procedure. The procedure was considered complete only when the entire uterine cavity was visualized. When abnormalities were observed, operative hysteroscopy was performed at the same time with diagnostic hysteroscopy. Cervical dilatation was performed before the resectoscope was inserted. The procedures were performed by 4 surgeons. The technique used varied depending on the uterine pathology found and the operating physician. Endometrial polyps, fibroids, endometrial hyperplasia, retained placental tissue and endometritis were confirmed by histology.

### 2.3. Variables

The primary outcome was hysteroscopic findings. As a second outcome parameter, we evaluated fertility outcomes after operative hysteroscopic procedure (number of pregnancies, births, miscarriages and ectopic pregnancies). The participant’s place of residence (urban areas or rural areas), age and type of infertility (primary infertility or secondary infertility) were also included in the analysis.

### 2.4. Data Sources 

The place of origin, age, diagnostics and hysteroscopic findings of the participants were collected from the medical reports stored in the hospital database. In addition, data on hysteroscopic procedures performed in patients with abnormal hysteroscopic findings were collected from operative registers stored in the clinic’s database.

### 2.5. Bias

To minimize bias, the selection of the participants was made without knowing the outcomes. The cases were consecutively collected. For preventing information bias, we used only hospital medical reports. The follow-up was carried out by telephone, the questions being the same for all participants in an identical format, and the answers were recorded in a uniform manner.

### 2.6. Statistical Methods

The data were stored in the Microsoft Office Excel (Microsoft Corporation, Redmond, WA, USA) software. Statistical analysis was performed using GraphPad Prism 8.0.2 (GraphPad Software, Inc., 2365 Northside Dr. Suite 560, San Diego, CA, USA). An amount of 95%CI was calculated for all proportion using Wilson–Brown method. Fisher’s exact test was used to compare the proportions. The probability of pregnancy was calculated by Kaplan–Meier analysis. *p* < 0.05 was considered significant.

## 3. Results

### 3.1. Participants Characteristics

A total of 198 women met the inclusion criteria and were included in the study, with 136 (68.7%; 95%CI = 61.9–74.7) from urban areas and 62 (31.3%; 95%CI = 25.3–38.1) from rural areas. The age of the participants varied between 23 years old and 47 years old, with a median of 34 years old (interquartile interval = 7). A total of 134 (67.7%; 95%CI = 60.9–73.8) women were diagnosed with primary infertility and 64 (32.3%; 95%CI = 26.2–39.1) with secondary infertility (Table 1). The participants’ follow-up was between 7 and 42 months, with a median of 25 months. Among the participants, in addition to infertility, abnormal uterine bleeding was found in 53 of the patients (26.8%, 95%CI = 21.1–33.3), pain in two patients (1.0%, 95%CI = 0.2–3.6) and amenorrhea in three patients (1.5%, 95%CI = 0.4–4.3).

### 3.2. Hysteroscopic Finding among Infertile Women

Among the 198 infertile women, 73 (36.9%, 95%CI = 30.5–43.8) had no uterine abnormalities on diagnostic hysteroscopy. 

The most common abnormalities found were endometrial polyps (*n* = 78; 39.4%, 95%CI = 32.9–46.3) and uterine synechiae (*n* = 21; 10.6%, 95%CI = 7.0–15.7) (Table 2). 

Endometrial polyps were found to be significantly more common in women with primary infertility compared to women with secondary infertility (OR = 5.18, *p* < 0.001). However, no statistically significant difference was found between the two subgroups (primary infertility vs. secondary infertility) in the other hysteroscopically detected uterine abnormalities (Table 2).

### 3.3. Fertility Outcomes after Operative Hysteroscopy

Operative hysteroscopy was performed in all patients with uterine abnormalities found. Endometrial resection was performed in patients with endometrial hyperplasia, all of whom had, in addition to infertility, abnormal uterine bleeding (*n* = 8/8). 

Reintervention was necessary in 3/78 (3.8%) cases of polypectomy, 2/9 (22.2%) cases of myomectomy, 1/7 (14.3%) cases of metroplasty and 4/21(19.0%) cases of resection of uterine synechia. 

A total of 42 patients (21.2%) conceived pregnancy after operative hysteroscopy. Of these, 34 (81.0%) of these pregnancies were conceived spontaneously and eight (19.0%) by IVF. The outcomes of pregnancies obtained through IVF were birth in 87.5% (*n* = 7/8) and miscarriage in 12.5% (*n* = 1/8).

The pregnancy rate in infertile women was 23.1%, 11.1%, 42.9%, 23.8% and 50% after polypectomy, myomectomy, metroplasty, synechiae resection and endometrial resection, respectively (Table 3). 

**Figure 1:**
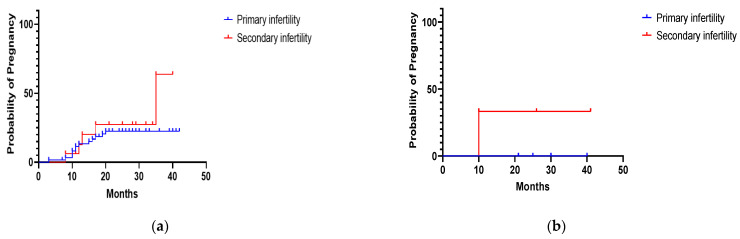
(**a**) Probability of pregnancy during the follow-up period in 78 infertile women who underwent hysteroscopic polypectomy. (**b**) Probability of pregnancy during the follow-up period in 9 infertile women who underwent hysteroscopic myomectomy.

**Figure 2:**
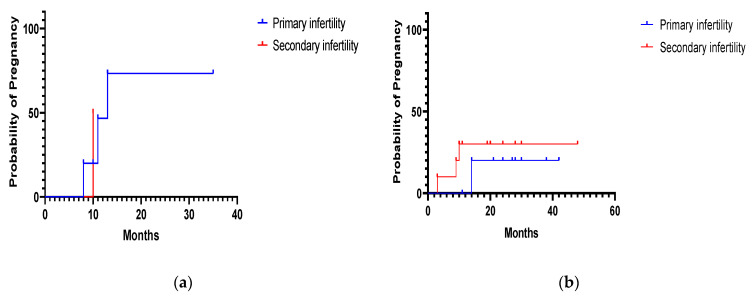
(**a**) Probability of pregnancy during the follow-up period in 7 infertile women who underwent hysteroscopic uterine septum resection. (**b**) Probability of pregnancy during the follow-up period in 21 infertile women who underwent hysteroscopic uterine synechiae resection.

No statistically significant differences were found between primary and secondary infertility in the pregnancy rate after polypectomy (*p* = 0.42), myomectomy (*p* = 0.15), septum resection (*p* = 0.55) or synechiae resection (*p* = 0.40) (Figure 1 and Figure 2).

## 4. Discussion

The World Health Organization (WHO) recognizes infertility as a public health issue worldwide, ranking it as the fifth largest severe disability in the young population [6].

In our study, of the 198 women diagnosed with infertility, 67.7% had primary infertility. Several studies worldwide have shown that the incidence of primary infertility between 57.5–69.5% is higher than that of secondary infertility [6,7,8,9]. However, in a study by Pansky et al., 221 infertile women showed a lower incidence of primary infertility (48%) compared to secondary infertility (52%) [10]. Depending on the women’s background, the Indian National Family Health Survey showed that the prevalence of primary infertility is higher compared to secondary infertility in urban areas. In our study among women in urban areas, the prevalence of primary infertility is higher than secondary infertility, while in women from rural areas, secondary infertility has a higher prevalence.

In the female, infertility may be caused by tubal disorders, uterine disorders, disorders of the ovaries or disorders of the endocrine system [1]. According to the WHO, “uterine disorders can be inflammatory in nature (i.e., endometriosis), congenital in nature (i.e., septate uterus) or benign in nature (i.e., fibroids) [1]. 

Considering that uterine lesions are common in infertile patients, being found in 34–62% of these patients, the evaluation of the uterine cavity is an important step during infertility work-up [10]. Hysteroscopy is considered the gold standard for evaluating the uterine cavity. The American College of Obstetricians and Gynecologists (ACOG) and the American Society for Reproductive Medicine (ASRM) recommend, during the basic assessment of infertility, imaging of the reproductive organs. However, they indicate hysteroscopy to confirm and treat intracavitary lesions detected by other imaging methods [5].

In our study, 36.9% of women who underwent infertility assessment had a normal uterine cavity on hysteroscopy. These results are lower than in other studies, which reported that 43% to 70% of infertile women have a normal uterine cavity [10]. The most common uterine abnormality diagnosed on hysteroscopy in the current study was uterine polyps (39.4%). The incidence of polyps is estimated to be between 3.8–38.5% in primary infertility, and between 1.8–17% in secondary infertility [11]. Our study also found a significant difference between the prevalence of endometrial polyps in women with primary infertility (31.3%) and those with secondary infertility (8.1%). Moreover, endometrial polyps are the most commonly reported uterine abnormalities diagnosed by hysteroscopy prior to in vitro fertilization (IVF) [12]. The gold standard in the diagnosis of endometrial polyps is hysteroscopy with guided biopsy [11]. Alone, hysteroscopy is more accurate than other imaging methods in diagnosing endometrial polyps, having a reported sensitivity of 58% to 99%, specificity of 87% to 100%, positive predictive value (PPV) of 21% to 100% and negative predictive value (NPV) of 66% to 99% [11]. 

Other abnormalities found on hysteroscopy in the current study were submucosal fibroids (4.5%), uterine septum (3.5%), uterine synechiae (10.6%), endometrial hyperplasia (4.0%), endometritis (0.5%) and placental tissue residue (0.5%). 

In other studies, uterine fibroids are detected in approximately 5–10% of infertile women, in 1 to 2.4% of cases being the only abnormality detected [13]. Hysteroscopy is considered the gold standard to diagnose intracavitary fibroids, with a reported sensitivity, specificity and predictive values of up to 100% [14]. 

Regarding the uterine septum, even if this pathology is not a primary factor for infertility, approximately 40% of its patients have infertility, obstetric complications and an increased incidence of recurrent miscarriages [15]. The prevalence of uterine septum in infertile women varies, but is estimated to be 3.9%, similar to that in our study [16]. 

Another pathology detected hysteroscopically, less common in patients who were included in our study, was endometritis. Some studies have shown that infertile women have a wide range of vascular changes in endometrial samples, associated with both endometritis and polyps. Thus, the authors suggest that it is possible that the vessel axis of functional polyps actually originates from the evolution of vascular changes associated with endometritis [17].

Although first-line investigative tools for uterine factors are a 2D transvaginal scan, hysterosalpingogram and saline infusion sonography, hysteroscopy is considered the gold standard for assessing the uterine cavity worldwide. A major advantage of hysteroscopy is that it allows the treatment of any detected intrauterine abnormalities. There are many studies reporting the benefits of operative hysteroscopy [18].

Several studies have found an association between polypectomy and improved spontaneous pregnancy rates. In our study, the pregnancy rate after polypectomy is 23%, which is lower compared to other studies, where it is between 50–78.3% [19,20]. In a systematic review, Jee BC and Jeong HG concluded that women with unexplained infertility, as well as infertile women who intend to undergo intrauterine insemination, may benefit from endometrial polypectomy, although the level of evidence is low. However, there is no strong evidence to support polypectomy increasing the pregnancy rate in women who intend to undergo in vitro fertilization [21].

For submucosal myomas (The International Federation of Gynecology and Obstetrics classification), transhysteroscopic removal is the standard approach, the pregnancy rates after the procedure varying from 16.7 to 76.9%, with a mean value of 45% [22].

Hysteroscopic resection of uterine septum is performed worldwide to improve reproductive outcomes. Several studies have reported an increase in pregnancy rates after metroplasty, ranging from 23% to 80.6%, which is comparable with our results [23]. 

The relevance of operative hysteroscopy on infertility remains uncertain, and pregnancy rates after these interventions vary greatly. An independent factor that affects these rates is certainly the age of the women. A decrease in fertility occurs with increasing age, and these changes are generally corroborated with a decreasing ovarian reserve [24]. Aging leads to decreased fertility by reducing the quality of oocytes and decreasing female hormones [25]. Some studies show that the fertility rate decreases from 400 pregnancies per 1000 women under the age of 30 to 100 per 1000 women aged 45 years or older [25]. Therefore, we can consider that the pregnancy rate after operative hysteroscopy is also influenced by the age of the women.

The current study has some limitations. The study includes all women who presented between January 2018 and December 2020 in our clinic for the evaluation of infertility, where diagnostic hysteroscopy was performed as the primary tool. No participant had previously undergone other non-routine investigations to assess infertility (e.g., laparoscopy, thrombophilia testing, karyotype, immunologic testing, etc.) and may have other pathologies associated with this disease. Moreover, the male factor was not evaluated. In addition, some uterine conditions, such as adenomyosis or non-submucosal fibroids, may go unnoticed on hysteroscopy. Furthermore, tubal patency evaluation by hysterosalpingography or laparoscopy was not performed in any patient prior to hysteroscopy. However, by ultrasound evaluation performed before hysteroscopy, pathologies such as ovarian insufficiency, hydrosalpinx or polycystic ovary syndrome were excluded.

Thus, the results of the study show that the most common uterine abnormalities can be diagnosed by hysteroscopy among all infertile women, not only those with a suspected uterine pathology. 

## 5. Conclusions

Endometrial polyps were the most common uterine abnormality found in women with unexplained infertility. Hysteroscopic interventions appeared to increase pregnancy rates and outcomes among these women.

## Figures and Tables

**Table 1:** Characteristics of 198 infertile women.

	Primary Infertility (No. of Patients = 134)	Secondary Infertility (No. of Patients = 64)
Median Age (Range)	34 (23–47)	34.5 (25–47)
Urban Area N/total (%; 95%CI)	94/134 (70.1%; 61.9–77.2)	42/64 (65.6%; 53.4–76.1)
Rural Area N/total (%; 95%CI)	40/134 (29.9%; 22.8–38.1)	22/64 (34.4%; 23.9–46.6)

**Table 2:** Hysteroscopic finding among 198 women with unexplained infertility.

Hysteroscopic Finding	Total No. (%; 95%CI)	Primary Infertility No. (%; 95%CI)	Secondary Infertility No. (%; 95%CI)	*p* Value OR (95%CI)
Normal uterine cavity	73 (36.9%; 30.5–43.8)	44 (22.2%; 17.0–28.5)	29 (14.6%; 10.4–20.2)	*p* = 0.06 1.66 (1.00–2.77)
Endometrial Polyp	78 (39.4%; 32.9–46.3)	62 (31.3%; 25.3–38.1)	16 (8.1%; 5.0–12.7)	*p* < 0.001 5.18 (2.92–9.26)
Uterine Septum	7 (3.5%; 1.7–7.1)	5 (2.5%; 1.1–5.8)	2 (1.0%; 0.2–3.6)	*p* = 0.44 2.53 (0.53–12.87)
Uterine Synechiae	21 (10.6%; 7.0–15.7)	11 (5.6%; 3.1–9.7)	10 (5.1%; 2.8–9.0)	*p* > 0.99 1.10 (0.47–2.65)
Uterine Fibroids	9 (4.5%; 2.4–8.4)	6 (3.0%; 1.4–6.5)	3 (1.5%; 0.4–4.4)	*p* = 0.50 2.03 (0.51–7.48)
Endometrial Hyperplasia	8 (4.0%; 2.1–7.8)	6 (3.0%; 1.4–6.5)	2 (1.0%; 0.2–3.6)	*p* = 0.28 3.06 (0.73–15.05)
Endometritis	1 (0.5%; 0.02–2.8)	—	1 (0.5%; 0.02–2.8)	*NA*
Retained placental tissue	1 (0.5%; 0.02–2.8)	—	1 (0.5%; 0.02–2.8)	*NA*

*NA* = not applicable.

**Table 3:** Pregnancy rate among women with unexplained infertility after operative hysteroscopy.

	Total *n*/total (%; 95%CI)	Primary Infertility *n*/total (%; 95%CI)	Secondary Infertility *n*/total (%; 95%CI)
After hysteroscopic polypectomy			
Pregnancies	18/78 (23.1%; 15.1–33.6)	13/62 (21.0%; 12.7–32.6)	5/16 (31.3%; 14.2–55.6)
Births	17/78 (21.8%; 14.1–32.2)	13/62 (21.0%; 12.7–32.6)	4/16 (25.0%; 10.2–49.5)
Miscarriages	1/78 (1.3%; 0.1–6.9)	—	1/16 (6.3%; 0.3–28.3)
After hysteroscopic myomectomy			
Pregnancies	1/9 (11.1%; 0.6–43.5)	—	1/3 (33.3%; 1.7–88.2)
Births	1/9 (11.1%; 0.6–43.5)	—	—
Miscarriages	—	—	—
After metroplasty			
Pregnancies	3/7 (42.9%; 15.8–75.0)	2/5 (40.0%; 7.1–76.9)	1/2 (50.0%; 2.6–96.4)
Births	2/7 (28.6%; 5.1–64.1)	1/5 (20.0%; 1.0–62.4)	1/2 (50.0%; 2.6–96.4)
Miscarriages	1/7 (14.3%; 0.7–51.3)	1/5 (20.0%; 1.0–62.4)	—
After synechiae resection			
Pregnancies	5/21 (23.8%; 10.6–45.1)	2/11 (18.2%; 3.2–47.7)	3/10 (30.0%; 10.8–60.3)
Births	3/21 (14.3%; 5.0–34.6)	2/11 (18.2%; 3.2–47.7)	1/10 (10.0%; 0.5–40.4)
Miscarriages	2/21 (9.5%; 1.7–28.9)	—	2/10 (20.0%; 3.6–51.0)
After endometrial resection			
Pregnancies	4/8 (50.0%; 21.5–78.5)	2/6 (33.3%; 5.9–70.0)	2/2 (100%; 17.8–100)
Births	2/8 (25.0%; 4.4–59.1)	2/6 (33.3%; 5.9–70.0)	—
Miscarriages	1/8 (12.5%; 0.6–47.1)	—	1/2 (50.0; 2.6–97.4)
Ectopic pregnancy	1/8 (12.5%; 0.6–47.1)	—	1/2 (50.0; 2.6–97.4)

## Data Availability

The data presented in this study are available on request from the corresponding author.
